# Annual wellness visits and care management before and after dialysis initiation

**DOI:** 10.1186/s12882-021-02368-0

**Published:** 2021-05-05

**Authors:** Virginia Wang, Lindsay Zepel, Clarissa J. Diamantidis, Valerie A. Smith, Sarah Hudson Scholle, Matthew L. Maciejewski

**Affiliations:** 1 Center of Innovation to Accelerate Discovery and Practice Transformation, Durham Veterans Affairs Health Care System, Durham, NC USA; 2 Department of Population Health Sciences, Duke University School of Medicine, Durham, NC USA; 3 Division of General Internal Medicine, Department of Medicine, Duke University School of Medicine, Durham, NC USA; 4 Division of Nephrology, Department of Medicine, Duke University School of Medicine, Durham, NC USA; 5 National Committee for Quality Assurance, Washington, DC USA; 6 OptumLabs Visiting Fellow, Cambridge, MA USA

**Keywords:** Medicare, Beneficiary, dialysis, Annual wellness visit, Prevention

## Abstract

**Introduction:**

Demands of dialysis regimens may pose challenges for primary care provider (PCP) engagement and timely preventive care. This is especially the case for patients initiating dialysis adjusting to new logistical challenges and management of symptoms and existing comorbid conditions. Since 2011, Medicare has provided coverage for annual wellness visits (AWV), which are primarily conducted by PCPs and may be useful for older adults undergoing dialysis.

**Methods:**

We used the OptumLabs® Data Warehouse to identify a cohort of 1,794 Medicare Advantage (MA) enrollees initiating dialysis in 2014–2017 and examined whether MA enrollees (1) were seen by a PCP during an outpatient visit and (2) received an AWV in the year following dialysis initiation.

**Results:**

In the year after initiating dialysis, 93 % of MA enrollees had an outpatient PCP visit but only 24 % received an annual wellness visit. MA enrollees were less likely to see a PCP if they had Charlson comorbidity scores between 0 and 5 than those with scores 6–9 (odds ratio (OR) = 0.59, 95 % CI: 0.37–0.95), but more likely if seen by a nephrologist (OR = 1.60, 95 % CI: 1.01–2.52) or a PCP (OR = 15.65, 95 % CI: 9.26–26.46) prior to initiation. Following dialysis initiation, 24 % of MA enrollees had an AWV. Hispanic MA enrollees were less likely (OR = 0.57, 95 % CI: 0.39–0.84) to have an AWV than White MA enrollees, but enrollees were more likely if they initiated peritoneal dialysis (OR = 1.54, 95 % CI: 1.07–2.23) or had an AWV in the year before dialysis initiation (OR = 4.96, 95 % CI: 3.88–6.34).

**Conclusions:**

AWVs are provided at low rates to MA enrollees initiating dialysis, particularly Hispanic enrollees, and represent a missed opportunity for better care management for patients with ESKD. Increasing patient awareness and provider provision of AWV use among dialysis patients may be needed, to realize better preventive care for dialysis patients.

## Introduction

Just as outpatient care from a nephrologist is recommended to improve patients’ transition to dialysis and outcomes, [1] ongoing care from a primary care provider (PCP) is important for dialysis patients [2]. However, there is limited evidence on what amount of PCP involvement in dialysis patient care is optimal. Central to building this evidence base is understanding patients’ actual patterns of care, including how patients initiating dialysis engage with PCPs. This is particularly important during the critical period just before and after initiating dialysis, when coordination between providers could help patients adjust to new logistical challenges and support management of symptoms and existing comorbid conditions. Annual wellness visits (AWVs) may be especially useful for older adults with end-stage kidney disease (ESKD) initiating dialysis because they often have multiple comorbid conditions that require management by PCPs, nephrologists and other specialists [3]. In contrast to an annual physical exam or regular care visits, an AWV is a patient visit with their physician to review and modify a personalized prevention plan based upon a health risk assessment, focused on preventing illness based on their current health and risk factors [4, 5]. Since 2011, Medicare has provided coverage for AWVs at no cost to beneficiaries to improve access and receipt of primary care management.

However, receipt of AWV among dialysis patients may be challenging, as the thrice-weekly dialysis treatment regimen may pose barriers to timely receipt of care outside the dialysis setting, especially for managing non-renal comorbid illness and complications. A literature review found that most dialysis patients rely on nephrologists for their primary care despite having a PCP and that care coordination between PCPs and nephrologists is suboptimal because of physician self-reported confusion about their roles [2]. A study of dialysis patients dually enrolled in Medicare and Medicaid public insurance programs in New Jersey in 1990–1996 found low rates of screening for prostate cancer, mammography, HbA1c and pap smears, suggesting that the quality of preventive care that could be delivered by AWV provision is suboptimal [6]. As a result, dialysis initiation raises the potential for care fragmentation that may exacerbate challenges to the receipt of timely preventive care, including AWVs that are most often provided by PCPs [7].

AWVs provide a unique opportunity outside of regular care visits to update care plans via a health risk assessment, screen for depression and functional limitations, and better coordinate care [4]. Although Medicare provides coverage for AWV at no cost to beneficiaries to encourage provision of preventive care, AWV uptake rates are low among patients enrolled in the Medicare fee-for-service (Medicare FFS) program (7.5 % in 2011 to 15.6 % in 2014) compared to patients in the Medicare Advantage (MA) program [7–9]. In contrast to the traditional Medicare FFS coverage of care through an open network of participating providers, the MA program furnishes services under Medicare-regulated private plans that manage inpatient and outpatient care. Both Medicare FFS and MA provide coverage for older adults and disabled individuals.

No studies have examined AWV rates in Medicare patients with CKD initiating dialysis who are particularly vulnerable to disruptions in preventive care. A recent non-randomized study found that patients participating in a dialysis-centric patient-centered medical home were more likely to see a PCP, particularly in the subset who had no established PCP relationship before enrollment [10]. MA care may be organized similarly to patient-centered care models under expansion, compared to that of Medicare FFS. Building from this earlier literature, this study examines the use of PCP care management and receipt of AWVs in Medicare Advantage (MA) enrollees initiating dialysis in 2014–2017. We also examine patient factors associated with care management by a PCP and receipt of AWVs to understand factors that explain variations in their provision. Findings of AWV use among MA patients with advanced CKD transitioning to dialysis may elucidate trends and opportunities that are relevant to ongoing expansions of MA and private sector comprehensive care models for ESKD [11].

## Materials and methods

### Data source and cohort

**Fig. 1:**
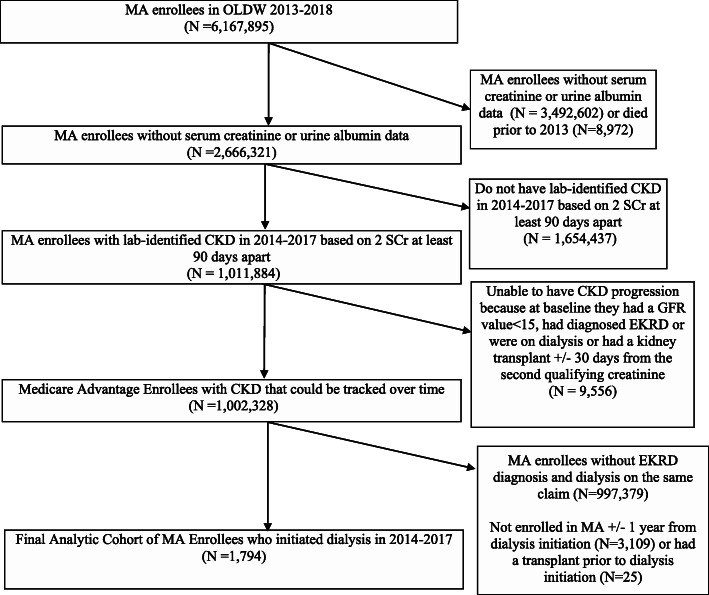
STROBE figure of analytic cohort

This study used de-identified administrative claims data from the OptumLabs® Data Warehouse (OLDW), which includes medical and pharmacy claims, laboratory results, and enrollment records for commercial and Medicare Advantage (MA) enrollees. The database contains longitudinal health information on enrollees and patients, representing a diverse mixture of ages, ethnicities and geographical regions across the United States [12]. We identified a cohort of MA enrollees with CKD and medical coverage during 1/1/2013 to 12/31/2018. The initial population of MA enrollees in 2013–2018 included 6,167,895 individuals (Fig. 1) who were then excluded if they died prior to 2013 (*n* = 8972), lacked serum creatinine or urine albumin lab values needed to identify CKD (*n* = 3,492,602) or did not have two serum creatinine values at least 90 days apart indicating CKD stages 1–5 (1,654,437). We identified 1,011,884 MA enrollees with lab-identified CKD stages 1–5 on the basis of two serum creatinine values at least 90 days apart with at least 1 urine albumin value with an albumin-to-creatinine ratio (UACR) ≥ 30 mg/g. Enrollees were excluded if they were unable to have CKD progression tracked because at baseline they had an glomerular filtration rate (GFR) value < 15 mL/min/1.73m^2^, had diagnosed ESKD or were on dialysis or had a kidney transplant +/-30 days of the second qualifying serum creatinine (*n* = 9,556).

We excluded an additional 997,379 MA enrollees who did not initiate dialysis during the study period, on the basis of not having an ESKD diagnosis and dialysis on the same claim. Last, we excluded MA enrollees not alive or continuously enrolled in MA a full year before and after dialysis initiation (n = 3,109) or those receiving a kidney transplant prior to dialysis initiation (N = 25). The final analytic sample included 1,794 MA enrollees who initiated dialysis in 2014–2017 and remained in Medicare Advantage the year preceding and following dialysis initiation.

### Outcome, Covariates and analysis

We had two outcomes of interest: visit with a PCP and receipt of AWV in the year following dialysis initiation. From OLDW data, we constructed a binary outcome of whether an enrollee had an outpatient visit with a PCP, based on claims with family practice or internal medicine provider specialty codes (e.g., family practice, internist, or internal medicine specialist) in outpatient settings where primary care providers may care for patients (i.e., physician’s office, urgent care facility, ambulatory surgical center, independent clinic, outpatient hospital, telehealth, federally qualified health center, state or local public health clinic, rural health clinic). Receipt of an AWV within the year following dialysis initiation was ascertained from Current Procedural Terminology (CPT) codes G0402, G0438, or G0439 in medical claims.

We also included several patient characteristics for covariate adjustment: age group at dialysis initiation (18–64, 65–74, 75+), sex, race/ethnicity (White, Hispanic, Black, Asian, unknown), geographic location (metropolitan) from Census tract-based Rural Urban Community Area (RUCA) codes, comorbidity burden in the year prior to dialysis initiation via the Charlson comorbidity index (0–5, 6–9, 10+) [13], initial dialysis modality, as well as indicators whether patients had an outpatient visit with a PCP or nephrologist, clinical recognition of CKD, and receipt of an annual wellness visit in the year prior to dialysis initiation. Outpatient visit provider types (PCP, nephrologist) were ascertained from provider specialty code in claims. An MA enrollee was indicated to have clinically recognized CKD if there was at least one diagnosis in the year prior to dialysis initiation, which was determined from inpatient and outpatient claims indicating CKD stages 1–5 (ICD-9 codes 585.1-585.5; ICD-10 codes N18.1-N18.5) or unknown stage (ICD-9 code 585.9; ICD-10 code N18.9).

Incident dialysis modality was ascertained from OLDW’s MA enrollee claims using CPT codes and revenue codes (CPT codes for hemodialysis (HD): 90935, 90937; peritoneal dialysis (PD): 90945, 90947; Unspecified: 90999; Revenue codes HD: 0820, 0821, 0825, 0829; PD: 0830, 0831, 0840, 0841, 0849, 0850, 0851, 0859). The most frequent CPT modality-specific code in the 1 month after dialysis initiation was used to assign patients to either HD or PD. For patients with MA claims for unspecified dialysis (the most commonly used code), we used frequency of unique treatment session to assign modality: 1) > 15 unique treatment days for unspecified dialysis was identified as PD (i.e., consistent with PD-specific CPT billing patterns), 2) 10–15 unique treatment session days corresponded to HD, and 3) modality for < 10 unique treatment session days of unspecified dialysis was determined by revenue code.

In descriptive analysis, patient characteristics were summarized using mean and standard deviation for continuous variables and frequency and percent for categorical variables. In adjusted analysis, we used logistic regressions to assess patient factors associated with seeing a PCP in the year after dialysis initiation and receipt of an AWV following dialysis initiation. To identify potential disparities in care, we also examined variation in the proportion of MA enrollees with an AWV by race/ethnicity, geography and outpatient provider types. The institutional review board approved the study.

## Results

### Characteristics of Analytic Cohort

The cohort of 1,794 MA enrollees had a mean (median) follow-up of 2.0 (1.8) years and an average age of 72.0 years at dialysis initiation (Table 1). Approximately half of the MA cohort was female (46.7 %), white race (48.9 %), the majority lived in metropolitan areas (88.4 %) and most initiated hemodialysis (90.4 %). The mean Charlson score for the cohort was 7.5, a vast majority (81.3 %) had diagnosed diabetes, nearly all (99.8 %) had diagnosed hypertension and clinical recognition of their CKD via diagnoses (97.7 %) already present in their records. The majority (79.2 %) saw a nephrologist during an outpatient visit in the year before dialysis initiation, either with or without co-management by a PCP (75.9 % vs. 3.2 %, respectively, results not shown).

**Table 1:** Descriptive statistics of medicare advantage enrollees initiating dialysis (2014-17)

	Overall Cohort (*N*=1,794)
Age (Mean, Standard Deviation)	72.0 (9.3)
Age Group (N, %)	
18-64	309 (17.2%)
65-74	744 (41.5%)
75+	741 (41.3%)
Sex (N, %)	
Female	838 (46.7%)
Male	956 (53.3%)
Race & Ethnicity (N, %)	
White	877 (48.9%)
Black	544 (30.3%)
Asian	63 (3.5%)
Hispanic	256 (14.3%)
Unknown	54 (3.0%)
Geographic Location (N, %)	
Metropolitan	1,585 (88.4%)
Non-Metropolitan	209 (11.6%)
Diabetes (N, %)	1,458 (81.3%)
Hypertension (N, %)	1,791 (99.8%)
Charlson Comorbidity Index (Mean, Standard Deviation)	7.5 (2.6)
Charlson Comorbidity Index Group (N, %)	
0-5	382 (21.3%)
6-9	1,090 (60.8%)
10+	322 (17.9%)
Outpatient visit in the year prior to dialysis initiation: Provider type (N, %)	
Primary Care Physician	1,719 (95.8%)
Nephrologist	1,420 (79.2%)
Clinical recognition of Chronic Kidney Disease via diagnoses in year before dialysis initiation (N, %)	1,753 (97.7%)
Dialysis modality	
Hemodialysis	1,622 (90.4%)
Peritoneal dialysis	172 (9.6%)
Annual wellness visit in year prior to dialysis initiation (N, %)	
Yes	419 (23.4%)
No	1375 (76.6%)
Year of dialysis initiation (N, %)	
2014	108 (6.0%)
2015	331 (18.5%)
2016	540 (30.1%)
2017	815 (45.4%)

### Patient factors associated with seeing a PCP in the year after Dialysis initiation

In the year after dialysis initiation, 93.3 % of MA enrollees saw a PCP during an outpatient visit (results not shown). The odds of being seen by a PCP did not vary by race and ethnicity, sex or geography. In logistic regression (Table 2), MA enrollees age 18–64 (odds ratio (OR) = 0.50, 95 % confidence interval (CI): 0.29–0.85) and enrollees with Charlson scores between 0 and 5 were less likely (OR = 0.59, 95 % CI: 0.37–0.95) to see a PCP in the year after dialysis initiation than enrollees age 65–74 and enrollees with Charlson scores between 6 and 9, respectively. Dialysis patients enrolled in MA were more likely to see a PCP after dialysis initiation if they had a PCP visit in the year before dialysis initiation, compared to those who did not see a PCP prior to dialysis (OR = 15.65, 95 % CI: 9.26–26.46). Pre-dialysis visit with a nephrologist was also associated with greater odds of a PCP visit in the year after starting dialysis (OR = 1.60, 95 % CI: 1.01–2.52) relative to those who did not see a nephrologist prior to initiating dialysis.

**Table 2:** Predictors of seeing a PCP within year after dialysis initiation from logistic regression

	Odds Ratio (95% CI)
Age Group	
18-64	0.50 (0.29, 0.85)
65-74	Reference
75+	0.78 (0.49, 1.23)
Sex	
Female	0.97 (0.64, 1.45)
Male	Reference
Race & Ethnicity	
White	Reference
Black	0.79 (0.50, 1.23)
Asian	0.46 (0.19, 1.12)
Hispanic	1.54 (0.74, 3.19)
Unknown	0.72 (0.24, 2.12)
Geographic location	
Metropolitan	Reference
Non-Metropolitan	0.97 (0.51, 1.86)
Charlson Comorbidity Index	
0-5	0.59 (0.37, 0.95)
6-9	Reference
10+	0.66 (0.39, 1.10)
Outpatient visit in the year prior to dialysis initiation	
Primary Care Physician (reference: no PCP)	15.65 (9.26, 26.46)
Nephrologist (reference: no nephrologist)	1.60 (1.01, 2.52)
Dialysis modality	
Hemodialysis	Reference
Peritoneal Dialysis	0.87 (0.44, 1.72)
Annual wellness visit in year before dialysis initiation	
Yes	1.65 (0.96, 2.86)
No	Reference
Year of dialysis initiation	0.95 (0.77, 1.19)
c-statistic	0.76
Sample Size	1,794

### Provision of annual Wellness Visits

**Fig. 2:**
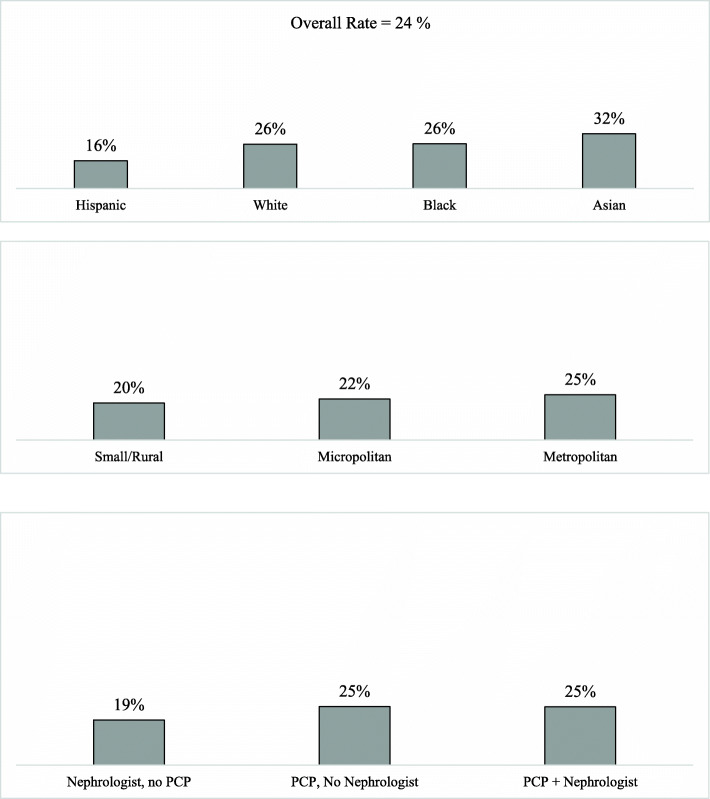
Unadjusted proportion of beneficiaries with an annual wellness visit in year of dialysis initiation, by race/ethnicity, geography and care management

In the year after dialysis initiation, 24 % of MA enrollees had an AWV (Fig. 2). Visit rates varied markedly by race and ethnicity (Fig. 2), with Hispanic enrollees being least likely (16 %) to have a visit and Asian enrollees most likely (32 %). There was little variability by urban geography (metropolitan) but some variation by the types of providers seen in the year prior to dialysis initiation. Enrollees who saw a nephrologist but not a PCP were least likely (19 %) to have an AWV, while enrollees who saw a PCP but not a nephrologist (25 %) or both a PCP and a nephrologist (25 %) were more likely to have an AWV in the year after dialysis initiation.

In the logistic regression (Table 3), Hispanic MA enrollees were less likely (OR = 0.57, 95 % CI: 0.39–0.84) to have an AWV than White enrollees, but Black and Asian enrollees were as likely to receive an AWV. Enrollees were more likely to have an AWV in the year after dialysis initiation if they initiated peritoneal dialysis (OR = 1.54, 95 % CI: 1.07–2.23), initiated dialysis later in the study period (OR = 1.20, 95 % CI: 1.05–1.38) or had an AWV in the year before dialysis initiation (OR = 4.96, 95 % CI: 3.88–6.34).

**Table 3:** Predictors of having an annual wellness visit within year after dialysis initiation from logistic regression

	Odds Ratio (95% CI)
Age Group	
18-64	0.83 (0.59, 1.18)
65-74	Reference
75+	0.91 (0.71, 1.18)
Sex	
Female	0.90 (0.71, 1.14)
Male	Reference
Race & Ethnicity	
White	Reference
Black	1.11 (0.85, 1.45)
Asian	1.25 (0.69, 2.25)
Hispanic	0.57 (0.39, 0.84)
Unknown	0.55 (0.26, 1.17)
Geographic Location	
Metropolitan	Reference
Non-Metropolitan	0.70 (0.48, 1.03)
Charlson Comorbidity Index	
0-5	1.02 (0.76, 1.36)
6-9	Reference
10+	0.82 (0.59, 1.13)
Outpatient visit in the year prior to dialysis initiation	
Primary Care Physician (reference: no PCP)	1.39 (0.74, 2.62)
Nephrologist (reference: no Nephrologist)	0.95 (0.72, 1.27)
Dialysis modality	
Hemodialysis	Reference
Peritoneal Dialysis	1.54 (1.07, 2.23)
Annual wellness visit in year before dialysis initiation	
Yes	4.96 (3.88, 6.34)
No	Reference
Year of dialysis initiation	1.20 (1.05, 1.38)
c-statistic	0.71
Sample Size	1,794

## Discussion

To our knowledge, this is the first study to examine the receipt of primary care among patients transitioning from CKD to ESKD and annual wellness visits in a cohort of Medicare Advantage enrollees initiating dialysis. These two outcomes were examined because prior studies have shown that AWVs are nearly exclusively provided by PCPs [7]. Our results indicate that most MA enrollees receive outpatient PCP care in the year after initiating dialysis, but fewer of these patients undergo an AWV during this same time period. Nearly all enrollees were seen by a PCP and a high proportion (79.2 %) were seen by a nephrologist prior to dialysis initiation, which suggests that MA enrollees were well integrated with these two types of providers. Patients were more likely to see a PCP in the year after dialysis initiation if they had seen a PCP or a nephrologist in the year prior, suggesting that patients continued to be engaged in care after the challenging transition to maintenance dialysis initiation.

AWV provision was suboptimal, especially for Hispanic enrollees. This finding is consistent with a prior study of MA enrollees and FFS beneficiaries in a large Northern California outpatient health system [9]. We also found that AWV provision was more likely among MA enrollees who had an AWV in the year before dialysis initiation, which is consistent with results from the general FFS population [7]. Nearly one-fourth (24 %) of MA enrollees had an AWV in the year after dialysis initiation, which is higher than the 15.6 % rate seen in the overall Medicare FFS population [7] and consistent with the AWV rate observed among MA enrollees in a prior study [9]. Given the greater vulnerability of MA enrollees following dialysis initiation, this rate of AWV provision is quite low and represents a significant missed opportunity for better care management among a majority of enrollees.

Examining patient engagement with PCPs and receipt of AWVs following dialysis initiation is an important and timely topic. The growing population of patients with ESKD in the US, the majority of whom are eligible for near-universal coverage through Medicare, has prompted Medicare efforts to promote better management of enrollees with ESKD. These efforts include not only AWVs, but also CMS demonstrations of ESKD-specific accountable care organizations [14], alternative payment models, and expanded eligibility for Medicare Advantage to patients with ESRD. Underlying these collective efforts is the anticipation that healthcare risk-bearing provider models will furnish better preventive care. Our results of generally high rates of PCP use suggest the promise of MA to furnish PCP care to chronic dialysis patients, which may be an important precondition for receipt of preventive and coordinated care as well as disease management. At the same time, however, only one quarter of MA enrollees received an annual wellness visit in the year after transitioning to dialysis. Although rates of AWV in MA are still higher than patients enrolled in the fragmented system of Medicare FFS, our findings raise concerns about the effectiveness of managed care models to improve primary care, prevention, and overall health of this high need, and high cost patient population new to dialysis. As these efforts unfold, it will be important to further promote AWVs and develop the evidence base and monitor these policy impacts on care models, performance, and patient outcomes.

There are several limitations that must be acknowledged. First, these results do not generalize beyond MA enrollees who initiated dialysis in 2014–2017. Second, we defined outcomes from an inclusive range of care settings, which generates an upper bound on AWV and PCP rates. Despite this, we AWVs were provided to a small fraction (24 %) of patients. Relatedly, OLDW data did not have granular information on identifying providers like advanced practitioners (primary care or nephrology), which undercounts care from these providers. Third, it is unclear whether and how often AWVs substituted for other preventive care visits or how provision of AWVs impacted clinical or economic outcomes of these vulnerable enrollees. Next, lab values to identify CKD were only available on a subset of MA enrollees.

Finally, a limitation of these results is the use of an imputed race/ethnicity variable that likely has measurement error. Use of race/ethnicity in these analyses has implications both in the assessment of estimated GFR and as a covariate for adjustment. The race/ethnicity results should not be over-interpreted as they require validation in future work.

## Conclusions

These finding suggest that AWVs are provided at suboptimal rates to vulnerable MA enrollees, particularly Hispanic enrollees initiating dialysis. Opportunities to increase patient awareness and provider provision of AWVs may be needed to realize better preventive care for dialysis patients.

## Data Availability

The data presented in this manuscript is under the possession of the Centers for Medicare and Medicaid Services (CMS) and cannot be publicly shared.

## Abbreviations

PCP: Primary care provider
AWVs: Annual wellness visits
MA: Medicare Advantage
ESKD: End-stage kidney disease
FFS: Fee-for-service
OLDW: OptumLabs^®^ Data Warehouse
GFR: Glomerular filtration rate
CPT: Current Procedural Terminology
HD: Hemodialysis
PD: Peritoneal dialysis
RUCA: Rural Urban Community Area
OR: Odds ratio
CI: Confidence interval
